# Transgenderism in studies on the health of older adults: a systematic review

**DOI:** 10.1590/S2237-96222024v33e2024304.especial.en

**Published:** 2025-01-10

**Authors:** Jônathas de Lima Arruda, Patrícia Fernanda Faccio, Camila Caroline da Silva, Danielle Ramalho Barbosa da Silva, Rafael da Silveira Moreira, Vanessa de Lima Silva

**Affiliations:** 1 Universidade Federal de Pernambuco, Programa de Pós-Graduação em Gerontologia, Recife, PE, Brasil; 2 Universidade Federal de Pernambuco, Programa de Pós-Graduação em Saúde da Criança e do Adolescente, Recife, PE, Brasil

**Keywords:** Personas mayores, Personas de 80 años o más, Travestismo, Personas transgénero, Salud, Older adults, Older Adults aged 80 years or older, Transvestism, Transgender People, Health

## Abstract

**Objective:**

To describe how transgenderism is studied in research on the health of older adults.

**Methods:**

This was a systematic literature review, with searches conducted in September 2022 across the LILACS, MEDLINE/ PubMed, Embase, Web of Science and Scopus databases. The articles were selected independently by two reviewers. The risk of bias was assessed using the JBI Critical Appraisal Tools and data synthesis followed the Entreq and Swim protocols.

**Results:**

A total of 15 studies were included, eight qualitative and seven quantitative, from 2014 to 2023. Most of them specifically analyzed the transgender population. The qualitative studies focused on individuals’ perceptions and experiences, health care planning and support networks. The quantitative studies addressed gender characterization, health status (including mental health), and associations with social determinants.

**Conclusion:**

Despite the different approaches, the studies addressed the subject in the context of accumulated stigmas and discrimination faced this population.

## Study contributions

**Main results:**

The aging experience of transgender people is shaped by transphobia, ageism, loneliness, fear of discrimination by caregivers, concerns about public policies, and the impact of prejudice and social exclusion on mental health.

**Implications for services:**

The diversity of gender identities and sexualities is also present among the elderly population. Healthcare professionals must be prepared to provide careful, responsible, comprehensive, and equitable care.

**Perspectives:**

There is an analytical gap on the topic in Latin American studies. Future research can contribute to the scientific debate and health systems, particularly in service provision and management, aiming to promote public policies.

## RESUMEN

**Objetivo:**

Descrever como a transgeneridade é estudada em pesquisas sobre a saúde do idoso.

**Métodos:**

Trata-se de revisão sistemática da literatura, com busca realizada em setembro de 2022, nas bases de dados Lilacs, Medline/Pubmed, Embase, Web Of Science e Scopus. Os artigos foram selecionados por pares, independentemente. O risco de viés se guiou pelo JBI Critical Appraisal Tools e a síntese de dados pelos protocolos Entreq e Swim.

**Resultado:**

Foram incluídos 15 estudos, oito qualitativos e sete quantitativos, de 2014 a 2023. A maioria deles analisou especificamente a população transgênero. Os estudos qualitativos focaram as percepções e vivências dos indivíduos e o planejamento de cuidados em saúde e rede de apoio. Os estudos quantitativos focaram a caracterização do gênero dos sujeitos, situação de saúde (incluindo a saúde mental) e associações com determinantes sociais. Conclusão: Apesar dos enfoques diferentes, os estudos abordaram a temática a partir do acúmulo de estigmas e discriminações dessa população.

## INTRODUCTION

Population aging is a global reality, although it occurs unevenly due to socioeconomic inequities. The elderly group consists of individuals aged 60 or over. This group is growing faster than other age groups, correlating with demographic and epidemiological transitions.^1,2^

Old age occupies a paradoxical position: it stands out as a phase of greater wisdom and experience, reflecting the multiplicity of aging experiences,^3^ but it is also subject to rejection and interventions as technological advancements aim to halt the natural changes in the body during this process.^4^ Aging has historically been burdened with prejudiced symbols, particularly those related to sexuality, which are socially invalidated and have long been the subject of few studies and reflections in academic circles.^5^

In contrast to the cis-heteronormative understanding of old age, LGBTQIAPN+ gerontology has emerged more recently as a field of knowledge and discourse that focuses its analyses on the multiplicity of aging experiences. This gerontology views itself as counter-hegemonic conceptions of aging.^6^

Academia often adopts a biologically deterministic view of sexuality in old age, associating this stage of life solely with functional decline. This distortion is more prevalent when discussing LGBTQIAPN+ aging, as the understanding of sexuality has historically been limited to reproduction.^8^ LGBTQIAPN+ older adults bear a significant stigma: that of old age, of sexual minorities, and that of gender identity.^9^ When these factors intersect, political, social, and economic vulnerabilities are amplified.^10^

Transgender is an umbrella term used for people who identify with a gender that differs from the one assigned at birth, whose gender expression does not conform to social expectations.^11^ Violence against LGBTQIAPN+ people is often intentional and characterized by moral and physical assaults or threats. It is motivated by homophobia, which, although understood as aversion to homosexuality and homosexuals, also targets bisexuals and transgender people, occurring in both private and public spaces, from families to communities.^12^

It is important to emphasize that elderly transgender people are disproportionately affected by social determinants at both personal and community levels, leading to profound health inequalities. Studies on the aging of transgender people tend to be part of generalized analyses of sexual and gender minorities, resulting in an analytical gap regarding the specificities of transgenderism in old age.^13^

The stigma surrounding transgender people is also reproduced in academic works, as articles frequently associate transsexuality with sexual themes, drug use and HIV.^14^ The specific concerns of aging transgender individuals include unique issues compared to heterosexual people, such as fear of rejection by family and adult children, transphobia, marginalization by gays and lesbians, and discrimination by cisgender service providers.^15^

Given the relevance of sexual and gender minorities and the potential intersections between transgenderism, aging and health, this review was developed in order to characterize how transgenderism is addressed in research on the health of older adults.

## METHOD

This was a systematic literature review, guided by the PRISMA 2020 Checklist. This review was registered with PROSPERO, under number CRD42022360075. The research question was: *How is the transgender theme studied in research on the health of older adults?*, and it was structured as follows.

Population: older adults

Outcome: transgenderism

Context: health

In September 2022, searches were conducted in pre-selected electronic databases: LILACS, MEDLINE/ PubMed, Embase, Scopus and Web of Science.

The following key, formulated with Mesh descriptors, was applied to each database. LILACS: (mh:(aged)) OR (mh:(aged, 80 and over)) AND (mh:(health)) AND (mh:(sexual and gender minorities)) OR (mh:(gay)) OR (mh:(lesbian)) OR (mh:(bisexual)) OR (mh:(transgender persons)) OR (mh:(transsexualism)) OR (mh:(homosexuality)) OR (mh:(lesbianism)) OR (mh:(queer)) MEDLINE/PubMed: (((aged[MeSH Terms]) OR (aged, 80 and over[MeSH Terms])) AND (health[MeSH Terms])) AND (((((((((sexual and gender minorities[MeSH Terms]) OR (gay[MeSH Terms])) OR (lesbian[MeSH Terms])) OR (bisexual[MeSH Terms])) OR (transgender persons[MeSH Terms])) OR (transsexualism[MeSH Terms])) OR (homosexuality[MeSH Terms])) OR (lesbianism[MeSH Terms])) OR (queer[MeSH Terms])). Embase: aged OR (aged, 80 and over) AND (health) AND (sexual and gender minorities) OR (gay) OR (lesbian) OR (bisexual) OR (transgender persons) OR (transsexualism) OR (homosexuality) OR (lesbianism) OR (queer). Scopus: (KEY (aged) OR KEY (aged, 80 AND over) AND KEY (health) AND KEY (sexual AND gender AND minorities) OR KEY (gay) OR KEY (lesbian) OR KEY (bisexual) OR KEY (transgender AND persons) OR KEY (transsexualism) OR KEY (homosexuality) OR KEY (lesbianism) OR KEY (queer)). Web Of Science: AK=(aged OR aged, 80 and over AND health AND sexual and gender minorities OR gay OR lesbian OR bisexual OR transgender persons OR transsexualism OR homosexuality OR lesbianism OR queer). No filters or limits were used in the search for articles.

The files obtained from each database were uploaded into Rayyan. This is a collaborative virtual platform for literature reviews, where duplicate studies were excluded. The study selection was performed in two phases: abstract screening and full-text reading, independently and blindly by two reviewers (JLA and PF). Discrepancies were resolved by a third reviewer (CS) through consensus meetings. Data synthesis from the selected articles was performed in an Excel spreadsheet. The original review protocol specified only including studies with a minimum age of 60 years. This inclusion criterion was adjusted to increase the number of available studies, providing a richer and more representative database for analysis.

The inclusion criteria adopted for the analysis of the texts were: the scientific article had to be original, the study population had to include people aged 50 or older, the focus of the study had to be health and the transgender theme had to be addressed. Articles not focused on the aging process or the reality of older adults were not considered for analysis. In order to reduce the risk of bias, no eligibility criteria related to publication language, year, country, or institution of origin were applied.

A pilot selection was conducted to confirm the inclusion and exclusion criteria. This selection included the abstracts of the first 100 articles, organized in alphabetical order by title. After the consensus meeting on this pilot study, the selection of abstracts and full-text articles continued.

Data from the articles included in this review were extracted using a data extraction protocol, organized in a table within Microsoft Excel software, with a description of the following topics: article identification (article title, authors, year of publication, language and country of origin), objective, study population (study population and age group studied), method (study design, data collection instrument, study period, sample size and study location) and results (study focus, health approach, approach to the transgender theme approach and study outcomes) and conclusion.

The risk of bias analysis was performed by a researcher using JBI Critical Appraisal Tools for cross-sectional qualitative and quantitative study designs.^16,17^ Data synthesis was performed by grouping according to study design. The following tools were used: Entreq Statment^18^ for qualitative studies and Swim Guidelines^19^ for quantitative studies. The results were expressed in boxes and tables.

## RESULTS

**Figure 1:**
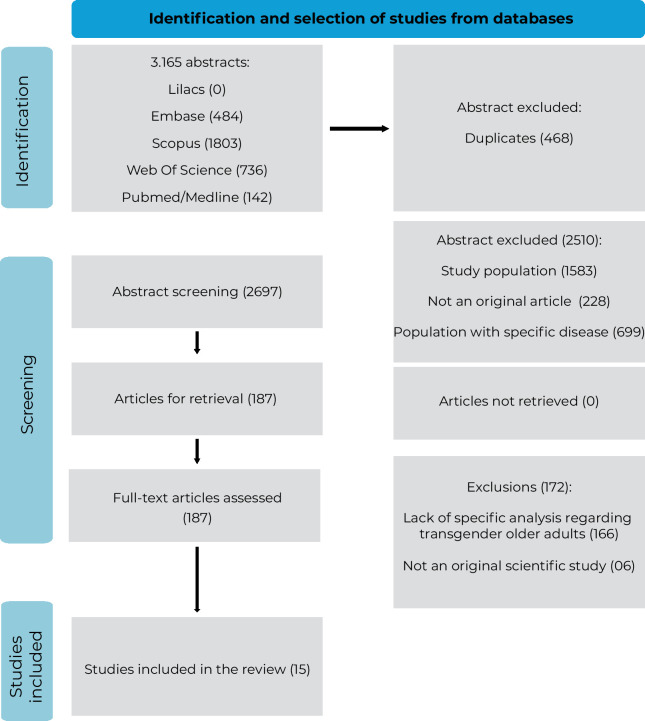
Study selection flowchart

A total of 3,165 abstracts were retrieved from the database searches, of which 484 were from Embase, 1,803 from Scopus, 736 from Web of Science and 142 from PubMed/MEDLINE. LILACS was the only database that returned no articles. After the exclusion of 468 duplicates, 2,697 files proceeded to the selection phase. A total of 2,510 studies were excluded at the abstract screening stage for not meeting the inclusion criteria, including: 1,583 where the population did not involve older adults, 228 that were not original articles and 699 included individuals with pre-existing conditions. A total of 187 articles were selected for full-text review, of which 172 were excluded. No articles were irretrievable during the selection process. The reasons for exclusion included the lack of specific analyses regarding transgender older adults in 166 articles, and 6 articles were not original studies. Ultimately, 15 articles were selected for this systematic review (Figure 1).

The analysis of the risk of bias in the qualitative studies indicated that all articles met the criteria proposed for the items evaluated, showing a low risk of bias. This pattern was identified in the quantitative cross-sectional studies, indicating a low risk of bias (Table 1).

**Box 1:** Analysis of the risk of bias of included studies

Analysis criteria										
**Joanna Briggs Critical Appraisal Tools – Qualitative Research**										
**Study**	**1**	**2**	**3**	**4**	**5**	**6**	**7**	**8**	**9**	**10**
Adan 2021^26^	Yes	Yes	Yes	Yes	Yes	Yes	No	Yes	Yes	Yes
Rosenwohl-Mack 2022^27^	Yes	Yes	Yes	Yes	Yes	Yes	No	Yes	Yes	Yes
Fabre 2014^28^	Yes	Yes	Yes	Yes	Yes	Yes	No	Yes	Yes	Yes
Pang, Gutman, Vries 2019^29^	Yes	Yes	Yes	Yes	Yes	Yes	Yes	Yes	Unclear	Yes
Muraco 2018^30^	Yes	Yes	Yes	Yes	Yes	Yes	Yes	Yes	Yes	Yes
Knochel, Flunker 2021^31^	Yes	Yes	Yes	Yes	Yes	Yes	No	Yes	Yes	Yes
Willis 2020^32^	Yes	Yes	Yes	Yes	Yes	Yes	Unclear	Yes	Yes	Yes
Page 2016^33^	Yes	Yes	Yes	Yes	Yes	Yes	Yes	Unclear	Yes	Yes
**Joanna Briggs Critical Appraisal Tools – Analytical Cross-Sectional Studies**										
**Study**	**11**	**12**	**13**	**14**	**15**	**16**	**17**	**18**		
Blosnich 2016^34^	Yes	Yes	Yes	Yes	Unclear	Unclear	Yes	Yes		
Hillman 2021^35^	Yes	Yes	Yes	Yes	Yes	Yes	Yes	Yes		
Today-Elis 2016^36^	Yes	Yes	Yes	Yes	Unclear	Unclear	Yes	Yes		
Nelson 2023^37^	Yes	Yes	Yes	Yes	Yes	Yes	Yes	Yes		
Bouman 2016^38^	Yes	Yes	Yes	Yes	Yes	Yes	Yes	Yes		
Fredriksen-Goldsen 2016^39^	Yes	Yes	Yes	Yes	Yes	Yes	Yes	Yes		
Today-Ellis 2022^40^	Yes	Yes	Yes	Yes	Yes	Yes	Yes	Yes		

a) Criteria for qualitative studies: (1) Is there congruence between the stated philosophical perspective and the research methodology? (2) Is there congruence between the research methodology and the research question or objectives? (3) Is there congruence between the research methodology and the methods used to collect data? (4) Is there congruence between the research methodology and the representation and analysis of the data? (5) Is there congruence between the research methodology and the interpretation of the results? (6) Is there a statement that locates the researcher culturally or theoretically? (7) Is the influence of the researcher on the research, and vice versa, addressed? (8) Are participants and their voices adequately represented? (9) Is the research ethical according to current standards, or for recent studies, is there evidence of ethical approval by an appropriate body? (10) Do the conclusions drawn in the research report flow from the analysis or interpretation of the data? b) Criteria for quantitative studies: (11) Were the inclusion criteria for the sample clearly defined? (12) Were the study subjects and setting described in detail? (13) Was the exposure measured in valid and reliable manner? (14) Were objective and standard criteria used for measuring the condition? (15) Were confounding factors identified? (16) Were strategies for dealing with confounding factors stated? (17) Were the outcomes measured in valid and reliable manner? (18) Was appropriate statistical analysis used?

Of the 15 studies included in this review, 13 were conducted in the United States, 1 in Canada and 1 in European countries (Belgium, Spain and the United Kingdom). The publication years range from 2014 to 2023. Of the total number of articles, 9 used 50 years or older as the age cutoff for the study population. The remaining articles used 55, 60, 61 and 65 years as the cut-off ages. One study included participants aged 21 to 70 years or olde**r.** Eight studies had female first authors (Table 1).

The qualitative studies mainly addressed life experiences and health perceptions and care planning for transgender older adults. They covered health care processes and the direction of public policies. Half of the studies focused on the lived experiences of the transgender population as the study’s object. The other half addressed the realities of the LGBTQIAPN+ population and gender diversity (Table 2).

**Table 1:** Characteristics of the studies included in the systematic review

Study	Country	Design	Age (years)	Population	Sample
Adan 2021^26^	United States	Qualitative	≥65	Transgender people	19
Fabbre 2014^28^	United States	Qualitative	≥50	Transgender people	22
Fredriksen-Goldsen 2016^39^	United States	Cross-Sectional	≥50	LGBT	4,627
Rosenwohl -Mack 2022^27^	United States	Qualitative	≥61	LGBTQIA+	21
Pang et al. 2019^29^	Canada	Qualitative	≥60	Transgender people	24
Knochel, Flunker 2021^31^	United States	Qualitative	≥55	Transgender people	24
Willis 2020^32^	United States	Qualitative	≥50	Transgender people	19
Muraco 2018^30^	United States	Qualitative	≥50	LGBT	59
Bouman 2016^38^	United Kingdom/Spain/Belgium	Cross-Sectional	≥50	Transgender people	71
Today-Ellis 2016^36^	United States	Cross-Sectional	≥50	Transgender people	186
Hillman 2021^35^	United States	Cross-Sectional	≥50	Transgender people	3,462
Blosnich 2016^34^	United States	Cross-Sectional	21-70+	Transgender people	6.307
Hol-Ellis et al. 2022^40^	United States	Cross-Sectional	≥50	LGBT	2,560
Nelson 2023^37^	United States	Cross-Sectional	≥50	LGBT	1,072
Page 2016^33^	United Kingdom	Qualitative	Not specified	Transgender people	Not specified

Among the qualitative studies included, the following stand out: the relationship between non- cisheteronormative users and healthcare professionals the implications of cultural, religious and moral values in the planning and execution of health care; the peculiarities of the LGBTQIAPN+ population in financial planning for old age; the importance of the social support network; and the specific challenges of transgender aging in the face of transphobia and ageism (Table 2).

The studies discussed specific actions aimed at optimizing the aging process for transgender people. These actions included providing trans-friendly housing, the need for healthcare professional training on gender diversity, care planning for transgender older adults (including non-human forms of support), and directing public actions, such as standardizing care and support processes.

Most of the quantitative studies addressed transgender identity as a means to characterize participants’ gender. These studies presented approaches related to the health status of the transgender older adults, with a focus on mental health. Studies on mental health examined the relationship between gender identity stigma and the correlation between hormone use and mental health (Table 3).

**Table 2:** Study of transgender themes in qualitative research

Study	Health approach	Transgenderism approach	Object of study	Study conclusion
Adan 2021^26^	Life experiences and health perceptions	Lived experience of transgender people is the object of study	Transgender people’s perspectives on health care and aging	Training for caregivers of transgender older adults is needed; fear of abuse and overlapping stigmas.
Fabbre 2014^28^	Life experiences and health perceptions	Lived experience of transgender people is the object of study	Gender transition among older adults	Professionals may perpetuate behaviors that invalidate queer aging.
Rosenwohl-Mack 2022^27^	Life experiences and health perceptions	Characterization of the gender of the subjects	Experience of older LGBTQIA+ individuals living in LGBTQ-friendly housing	The need to provide LGBTQ-friendly housing for older adults.
Pang 2019^29^	Care planning	Characterization of the gender of the subjects	Life Care Planning for Older LGBT Adults	Plan care for older adults and ensure that transgender aging is discussed.
Knochel, Flunker 2021^31^	Care planning	The lived experience of transgender people is the object of study	Care planning in old age for transgender and non-binary people	Early aging and the need to create institutionalized long-term transgender and non-binary older adults’ friendly care.
Willis 2020^32^	Healthcare	Gender characterization of subjects	Interaction with health professionals	Improve professionals’ knowledge on the topic of gender diversity, so that they do not reinforce gender-based inequalities by reiterating cisheteronormativity.
Muraco 2018^30^	Healthcare	Gender characterization of subjects	social support from animals among LGBT older adults	Various forms of support, including non-human support, can impact LGBT aging, especially for those with limited social networks.
Page 2016^33^	Public policies	The lived experience of transgender people is the object of study	Social participation of transgender older adults in health councils	Actions were defined to establish care standards for transgender individuals by mental health teams and implement a support process for transgender workers.

**Table 3:** Study of transgender themes in quantitative research

Study	Health approach	Transgender identity approach	Object of study	Conclusion of the study
Bouman 2016^38^	Mental health analysis	Characterization of the gender of the subjects	Correlation between hormone use and mental health	The use of cross-sex hormones before seeking medical treatment is widespread among older transgender women and appears to be associated with psychological benefits.
Today-Ellis 2016^36^	Mental health analysis	Characterization of the gender of the subjects; higher rates of gender dysphoria	Gender identity stigma and its correlation with mental health	By identifying the role of military service in the mental health of transgender older adults, this study provides insights into how prior military service may contribute to resilience and positive mental health outcomes.
Blosnich 2016^34^	Health analysis	Gender characterization of subjects based on ICD-9 diagnosis records	Prevalence of social determinants of health among transgender veterans and their associations with medical conditions	Social determinants play an important role in the lives of transgender people; hence the need to record them electronically and include them in treatment goals.
Hoy-Ellis 2022^40^	Health analysis	Characterization of the gender of the subjects	Differences in health exams, based on gender identity	Increasing access to preventive healthcare for transgender older adults through screening tests is essential to reducing health problems in this older adult population.
Fredriksen- Goldsen 2016^39^	Life experiences and health perceptions	Characterization of the gender of the subjects	Life events and their association with the well-being in older adults	Historical and environmental contexts frame normative and non-normative life events.
Hillman 2021^35^	Health analysis	The lived experience of transgender people is the object of study	Intimate partner violence against transgender older adults	Intimate partner violence is linked to poorer health outcomes. The study recommends surveillance that acknowledges gender identity and screening for abuse in individuals aged 50 and older.
Nelson 2023^37^	Public policies	Characterization of the gender of the subjects	Association between public policies and the health of older lesbians, gays, bisexuals and transgender people	Adult lesbian, gay, bisexual, and transgender are at a significantly higher risk of health problems if they live in a state with fewer enacted LGBT anti-discrimination policies.

The influence of social determinants on the health of transgender older adults, health status assessment, the impact of discrimination and hormone therapy on mental health, intimate partner violence and the relevance of public policies in combating transphobia were studied (Table 3). No missing or unclear data were identified in the studies included in this review.

## DISCUSSION

Scientific production on the health of transgender older adults presented a notable predominance of North American studies. Several factors have been listed to justify the disparities in scientific output between developed capitalist countries and middle- and low-income nations. These factors included: inequalities in science funding, the stronger presence of academic productivity logic in wealthier countries, the limited capture of locally focused research by international databases, and the possibility of editorial bias.^20^

An elderly person is defined as any individual aged 60 or older.^21^ There is disagreement among authors when studying the phenomenon of aging among sexual and gender minorities. Most studies use 50 years or older as the cut-off point, ten years younger than that established by the World Health Organization. Old age, marked by chronological demarcation, is a social construct, and the problematization of the age threshold should take into account locally relevant factors in each nation.^22^ Life experiences marked by social exclusion and discrimination, especially among transgender people, have direct impacts on quality of life and, consequently, on aging. The particularities of aging among sexual and gender minorities are permeated by discrimination and invisibility.^23^

One of the studies used the diagnosis of gender disorder as its basis, defined by the relevant code from the International Classification of Diseases, which is included in the health records of the target population. Transsexuality, as a reflection of the hierarchy of knowledge-power structures, reaffirming hegemonic gender norms, has historically been treated as a deviation, sometimes of a mental nature, sometimes of a sexual nature, as demonstrated by the shifting categories in the Diagnostic and Statistical Manual of Mental Disorders and the International Classification of Diseases. Medical, legal, psychiatric and psychological knowledge consciously pathologizes transgender people in order to classify, diagnose and intervene in their bodies.^24^

Western society is historically constructed and continually reinforcing the cis-heterosexual-patriarchal order, permeates social relations, which are characterized by various forms of violence, stigma, and discrimination against the transgender population. The incessant reproduction of gender and sex performance models results in exclusionary behaviors toward transgender individuals.^25^ The findings of this review reiterate that the cis-heterosexual order of performing gender and sexuality permeates the lived experiences of transgender people. This is reflected in difficulties in maintaining social support networks and accessing qualified care that addresses the specificities of their aging process. Transphobic behaviors persist throughout adult life. In old age, they are compounded by factors characteristic of the older adult population, such as family exclusion and fear of discriminatory behavior by caregivers and other residents in long-term care facilities.

The heterogeneity of the included studies, with different designs, which made it difficult to generalize the results, stands out as a limiting factor of this review.

The transgender theme in research on the health of older adults has been studied from the perspective of the accumulation of stigmas and the continuity of discrimination and exclusion during old age. Notable concerns include fear of loneliness, persistent transphobia, and the limited availability of open and welcoming spaces for transgender elderly individuals, often resulting in a more fragile social support network.

The importance of this review is justified by the social relevance of the topic, given the current scenario of population aging. Old age that contradicts cis-heteronormativity will require healthcare professionals and systems to understand the particularities and lived experiences of LGBTQIAPN+ older adults, especially transgender older adults. The lack of studies that correlate aging with sexual and gender minorities in Latin America and the Caribbean provides an opportunity for a contextualized analysis of the experiences of transgender older adults in the region.
