# Supplementary material for: Transgenderism in studies on the health of older adults: a systematic review
Source: Epidemiol Serv Saude. 2025 Jan 10;33(spe1):e2024304. doi: 10.1590/S2237-96222024v33e2024304.especial.en (PMC11734652; doi:10.1590/S2237-96222024v33e2024304.especial.en)
Supplement: Translation

# Translation: A transgeneridade em estudos sobre a saúde do idoso: revisão sistemática

**DOI:** 10.1590/S2237-96222024v33e2024304.especial.pt

**Authors:** Jônathas de Lima Arruda, Patrícia Fernanda Faccio, Camila Caroline da Silva, Danielle Ramalho Barbosa da Silva, Rafael da Silveira Moreira, Vanessa de Lima Silva

**Affiliations:** 1 Universidade Federal de Pernambuco, Programa de Pós-Graduação em Gerontologia, Recife, PE, Brasil; 2 Universidade Federal de Pernambuco, Programa de Pós-Graduação em Saúde da Criança e do Adolescente, Recife, PE, Brasil

**Keywords:** Idoso, Idoso de 80 Anos ou Mais, Travestilidade, Pessoas Transgênero, Saúde

## Abstract

**Objetivo:**

Descrever como a transgeneridade é estudada em pesquisas sobre a saúde do idoso.

**Métodos:**

Trata-se de revisão sistemática da literatura, com busca realizada em setembro de 2022, nas bases de dados Lilacs, Medline/Pubmed, Embase, Web Of Science e Scopus. Os artigos foram selecionados por pares, independentemente. O risco de viés se guiou pelo JBI Critical Appraisal Tools e a síntese de dados pelos protocolos Entreq e Swim.

**Resultado:**

Foram incluídos 15 estudos, oito qualitativos e sete quantitativos, de 2014 a 2023. A maioria deles analisou especificamente a população transgênero. Os estudos qualitativos focaram as percepções e vivências dos indivíduos e o planejamento de cuidados em saúde e rede de apoio. Os estudos quantitativos focaram a caracterização do gênero dos sujeitos, situação de saúde (incluindo a saúde mental) e associações com determinantes sociais.

**Conclusão:**

Apesar dos enfoques diferentes, os estudos abordaram a temática a partir do acúmulo de estigmas e discriminações dessa população.

## Contribuições do estudo

**Principais resultados:**

A velhice de pessoas transgênero é marcada por transfobia, idadismo, solidão, medo de sofrer discriminação por parte de cuidadores, preocupações relativas às políticas públicas e ao impacto do preconceito e exclusão social na saúde mental.

**Implicações para os serviços:**

As pluralidades relativas aos gêneros e às sexualidades também fazem parte da realidade da população idosa. Profissionais de saúde devem estar preparados para realizar o atendimento cuidadoso, responsável, integral e equânime.

**Perspectivas:**

Há lacuna analítica do tema em produções latino-americanas. Investigações futuras podem contribuir no debate científico e nos sistemas de saúde, no âmbito da assistência e gestão, buscando promover políticas públicas.

## INTRODUÇÃO

O envelhecimento populacional é uma realidade mundial, apesar de ocorrer de forma desigual, resultado das iniquidades socioeconômicas. O grupo de idosos é constituído por indivíduos com 60 anos de idade ou mais. Tal grupo cresce mais rápido do que as demais faixas etárias, correlacionando-se às transições demográfica e epidemiológica.^1,2^

A velhice ocupa lugar paradoxal: destaca-se como fase de maior sabedoria e experiências, refletindo a multiplicidade de velhices,^3^ mas também é objeto de rejeição e intervenções à medida que o aprimoramento tecnológico visa deter as mudanças naturais do corpo nesse processo.^4^ O envelhecimento é historicamente carregado de simbologias preconceituosas, em particular os aspectos relativos à sexualidade. Estes são socialmente invalidados e, por muito tempo, foram objeto de poucos estudos e reflexões em meio acadêmico.^5^

Em contraposição à compreensão cisheteronormativa da velhice, a gerontologia LGBTQIAPN+ surge mais recentemente enquanto campo de saberes e discursos que centra suas análises nas multiplicidades de experiências do envelhecimento. Essa gerontologia compreende-se como contra-hegemônica das velhices.^6^

A academia possui visão biologicista quanto à sexualidade na velhice, pois associa essa fase da vida unicamente ao declínio funcional. Tal distorção é mais presente ao tratar-se das velhices LGBTQIAPN+, já que a compreensão da sexualidade historicamente limita-se à reprodução.^8^ Idosos LGBTQIAPN+ sofrem grande carga de estigma: o da velhice, o das minorias sexuais e o de gênero.^9^ Ao interrelacioná-los, vulnerabilidades políticas, sociais e econômicas são potencializadas.^10^

Transgênero é um termo guarda-chuva usado para pessoas que se identificam com o gênero destoante daquele conferido ao nascimento, cuja expressão de gênero não está em conformidade com as expectativas sociais.^11^ As violências contra as pessoas LGBTQIAPN+ são frequentemente intencionais e caracterizadas por agressões moral e física ou por ameaças. Motivam-se pela homofobia, que, embora compreendida como a aversão a homossexualidade e homossexuais, alcança bissexuais e transgêneros, ocupando espaços privados e públicos, desde a família às comunidades.^12^

Cabe enfatizar que idosos transgêneros são desproporcionalmente afetados por determinantes sociais nos níveis pessoal e comunitário, desenhando-se quadros de profundas desigualdades em saúde. Estudos sobre o envelhecimento de pessoas transgêneros costumam estar nas análises generalizadas das minorias sexuais e de gênero, ocasionando vácuo analítico sobre as especificidades da transgeneridade na velhice.^13^

O estigma acerca de pessoas transgênero também é reproduzido nos trabalhos acadêmicos, pois é frequente a produção de artigos que associam transexualidade às temáticas sexuais, uso de drogas e HIV.^14^ As especificidades da velhice de pessoas transexuais englobam preocupações particulares, quando comparada à de pessoas heterossexuais, como medo de rejeição por parte da família e filhos adultos, transfobia, marginalização por parte de gays e lésbicas, além da discriminação por prestadores de serviço cisgênero.^15^

Diante da relevância que possui a temática das minorias sexuais e de gênero e as interlocuções possíveis entre transgeneridade, envelhecimento e saúde, esta revisão foi desenvolvida com o objetivo de caracterizar como a transgeneridade é abordada em pesquisas sobre a saúde de idosos. 

## MÉTODO

Trata-se de revisão sistemática da literatura, guiada pelo PRISMA 2020 Checklist. Esta revisão foi registrada junto à PROSPERO, sob o número CRD42022360075. A pergunta de pesquisa foi: *Como a temática transgênero é estudada em pesquisas sobre a saúde da pessoa idosa?*, estruturando-se conforme a seguir. 

População: idosos

Desfecho: transgeneridade

Contexto: saúde

Em setembro de 2022, realizou-se a busca em bases eletrônicas de dados previamente selecionadas: Lilacs, Medline/Pubmed, Embase, Scopus e Web of Science.

A seguinte chave, formulada com descritores do Mesh, foi aplicada em cada uma das bases. Lilacs: (mh:(aged)) OR (mh:(aged, 80 and over)) AND (mh:(health)) AND (mh:(sexual and gender minorities)) OR (mh:(gay)) OR (mh:(lesbian)) OR (mh:(bisexual)) OR (mh:(transgender persons)) OR (mh:(transsexualism)) OR (mh:(homosexuality)) OR (mh:(lesbianism)) OR (mh:(queer)) Medline/Pubmed: (((aged[MeSH Terms]) OR (aged, 80 and over[MeSH Terms])) AND (health[MeSH Terms])) AND (((((((((sexual and gender minorities[MeSH Terms]) OR (gay[MeSH Terms])) OR (lesbian[MeSH Terms])) OR (bisexual[MeSH Terms])) OR (transgender persons[MeSH Terms])) OR (transsexualism[MeSH Terms])) OR (homosexuality[MeSH Terms])) OR (lesbianism[MeSH Terms])) OR (queer[MeSH Terms])). Embase: aged OR (aged, 80 and over) AND (health) AND (sexual and gender minorities) OR (gay) OR (lesbian) OR (bisexual) OR (transgender persons) OR (transsexualism) OR (homosexuality) OR (lesbianism) OR (queer). Scopus: (KEY (aged) OR KEY (aged, 80 AND over) AND KEY (health) AND KEY (sexual AND gender AND minorities) OR KEY (gay) OR KEY (lesbian) OR KEY (bisexual) OR KEY (transgender AND persons) OR KEY (transsexualism) OR KEY (homosexuality) OR KEY (lesbianism) OR KEY (queer)). Web Of Science: AK=(aged OR aged, 80 and over AND health AND sexual and gender minorities OR gay OR lesbian OR bisexual OR transgender persons OR transsexualism OR homosexuality OR lesbianism OR queer). Não foram utilizados filtros ou limites na busca de artigos.

 Os arquivos obtidos a partir de cada uma das bases foram inseridos, por meio de *upload*, no Rayyan. Esta é uma plataforma virtual colaborativa para revisões de literatura, local de realização da exclusão de duplicidade de estudos. A seleção de estudos foi realizada em duas fases: leitura de resumos e leitura de artigos na íntegra, de forma independente cega por dois leitores (JLA e PF). As divergências foram resolvidas por um terceiro leitor (CS) em reunião de consenso. A síntese dos dados dos artigos selecionados foi realizada em planilha de Excel. No protocolo da revisão, havia previsão de somente incluir estudos com a idade mínima de 60 anos. Esse critério de inclusão foi modificado para aumentar o número de estudos disponíveis, proporcionando base de dados mais rica e representativa para a análise.

Os critérios de inclusão adotados para a análise dos textos foram: o artigo científico tinha que ser original, a população do estudo necessariamente deveria conter pessoas a partir dos 50 anos de idade, o objeto do estudo deveria ser a saúde e a temática transgênero deveria ser abordada. Os artigos cujo objeto de estudo não fosse voltado ao processo de envelhecimento ou à realidade de idosos não foram considerados para a análise. A fim de reduzir o risco de viés, não foram adotados, enquanto elegibilidade, critérios relativos ao idioma da publicação, ano, país ou instituição de origem.

Uma seleção piloto foi realizada com vistas à confirmação dos critérios de inclusão e exclusão. Nessa seleção, havia os resumos dos 100 primeiros artigos, organizados em ordem alfabética do título. Após a reunião de consenso desse estudo piloto, deu-se continuidade à seleção de resumos e à seleção de artigos na íntegra.

Os dados dos artigos incluídos na revisão foram extraídos de protocolo de extração de dados, organizado em tabela no *software* Microsoft Excel, com a descrição dos seguintes tópicos: identificação do artigo (título do artigo, autores, ano de publicação, idioma e país de origem), objetivo, população de estudo (população de estudo e idade estudada), método (desenho do estudo, instrumento de coleta, período do estudo, tamanho da amostra e local do estudo) e resultados (objeto do estudo, abordagem da saúde, abordagem da temática transgênero e resultado do estudo) e conclusão.

A análise do risco de viés foi realizada por um pesquisador por meio do JBI Critical Appraisal Tools para os desenhos de estudo qualitativos e quantitativos seccionais.^16,17^ A síntese dos dados foi feita a partir do agrupamento segundo o desenho de estudo. Utilizaram-se **o** Entreq Statment^18^ para os estudos qualitativos e as Diretrizes Swim^19^ para os estudos quantitativos. Os resultados foram expressos em quadros e tabela.

## RESULTADOS

Foram obtidos 3.165 resumos na busca em bases de dados, dos quais 484 foram oriundos da Embase, 1.803 da Scopus, 736 da Web Of Science e 142 da Pubmed/Medline. A Lilacs foi a única base consultada a não retornar artigos. Após a exclusão de 468 duplicatas, 2.697 arquivos prosseguiram na seleção. Foram excluídos 2.510 estudos na fase de leitura de resumos, por não se enquadrarem nos critérios de inclusão, entre os quais: 1.583 cuja população do estudo não envolveu idosos, 228 não eram artigos originais e 699 incluíam público com doença preexistente. Foram selecionados 187 arquivos para a leitura de artigos completos, dos quais 172 foram excluídos. Não houve artigos não recuperados durante a seleção. Entre os motivos das exclusões, 166 artigos não apresentaram análises específicas sobre idosos transgêneros e 6 não eram artigos originais. Ao final, 15 artigos foram selecionados para esta revisão sistemática (Figura 1).

**Figura 1:** Fluxograma de seleção dos estudos

A análise do risco de viés dos estudos qualitativos apontou que todos os artigos atenderam aos critérios propostos nos itens avaliados, o que representou baixo risco de viés. Tal padrão foi identificado nos estudos quantitativos do tipo seccional, assinalando o baixo risco de viés (Quadro 1).

**Quadro 1:** Análise do risco de viés dos estudos incluídos

Critérios de análise										
**Joanna Briggs Critical Appraisal Tools – Qualitative Research**										
**Estudo**	**1**	**2**	**3**	**4**	**5**	**6**	**7**	**8**	**9**	**10**
Adan 2021^26^	Sim	Sim	Sim	Sim	Sim	Sim	Não	Sim	Sim	Sim
Rosenwohl-Mack 2022^27^	Sim	Sim	Sim	Sim	Sim	Sim	Não	Sim	Sim	Sim
Fabre 2014^28^	Sim	Sim	Sim	Sim	Sim	Sim	Não	Sim	Sim	Sim
Pang, Gutman, Vries 2019^29^	Sim	Sim	Sim	Sim	Sim	Sim	Sim	Sim	Pouco claro	Sim
Muraco 2018^30^	Sim	Sim	Sim	Sim	Sim	Sim	Sim	Sim	Sim	Sim
Knochel, Flunker 2021^31^	Sim	Sim	Sim	Sim	Sim	Sim	Não	Sim	Sim	Sim
Willis 2020^32^	Sim	Sim	Sim	Sim	Sim	Sim	Pouco claro	Sim	Sim	Sim
Page 2016^33^	Sim	Sim	Sim	Sim	Sim	Sim	Sim	Pouco claro	Sim	Sim
**Joanna Briggs Critical Appraisal Tools – Analytical Cross Sectional Studies**										
**Estudo**	**11**	**12**	**13**	**14**	**15**	**16**	**17**	**18**		
Blosnich 2016^34^	Sim	Sim	Sim	Sim	Pouco claro	Pouco claro	Sim	Sim		
Hillman 2021^35^	Sim	Sim	Sim	Sim	Sim	Sim	Sim	Sim		
Hoy-Elis 2016^36^	Sim	Sim	Sim	Sim	Pouco claro	Pouco claro	Sim	Sim		
Nelson 2023^37^	Sim	Sim	Sim	Sim	Sim	Sim	Sim	Sim		
Bouman 2016^38^	Sim	Sim	Sim	Sim	Sim	Sim	Sim	Sim		
Fredriksen-Goldsen 2016^39^	Sim	Sim	Sim	Sim	Sim	Sim	Sim	Sim		
Hoy-Ellis 2022^40^	Sim	Sim	Sim	Sim	Sim	Sim	Sim	Sim		

a) Critérios para estudos qualitativos: (1) Existe congruência entre a perspectiva filosófica declarada e a metodologia de pesquisa? (2) Existe congruência entre a metodologia de pesquisa e a questão ou objetivos da pesquisa? (3) Existe congruência entre a metodologia de pesquisa e os métodos usados para coletar dados? (4) Existe congruência entre a metodologia de pesquisa e a representação e análise dos dados? (5) Existe congruência entre a metodologia de pesquisa e a interpretação dos resultados? (6) Existe uma declaração que localiza o pesquisador cultural ou teoricamente? (7) A influência do pesquisador na pesquisa, e vice-versa, é abordada? (8) Os participantes e suas vozes estão adequadamente representados? (9) A pesquisa é ética de acordo com os critérios atuais, ou para estudos recentes, e há evidências de aprovação ética por um órgão apropriado? (10) As conclusões tiradas no relatório de pesquisa decorrem da análise ou interpretação dos dados? b) Critérios para estudos quantitativos: (11) Os critérios de inclusão na amostra foram claramente definidos? (12) Os sujeitos do estudo e o cenário foram descritos em detalhes? (13) A exposição foi medida de forma válida e confiável? (14) Foram usados critérios objetivos e padrão para a medição da condição? (15) Foram identificados fatores de confusão? (16) As estratégias para lidar com os fatores de confusão foram declaradas? (17) Os resultados foram medidos de forma válida e confiável? (18) Foi utilizada análise estatística apropriada?

Dos 15 estudos que integraram esta revisão, 13 foram desenvolvidos nos Estados Unidos, 1 no Canadá e 1 em países europeus (Bélgica, Espanha e Reino Unido). Os anos das publicações variam de 2014 a 2023. Do total de artigos, 9 utilizaram como ponto de corte para o público estudado a idade igual ou superior a 50 anos. Os demais artigos adotaram as idades igual ou superior a 55, 60, 61 e 65 anos como ponto de corte. Um artigo trabalhou com o público cujas idades variaram de 21 a 70 anos de idade ou mais. Oito artigos tiveram nomes femininos citados como primeiro autor (Tabela 1).

As temáticas abordadas nos estudos qualitativos voltaram-se, em sua maioria, às experiências de vida e percepções de saúde e ao planejamento de cuidados de idosos transgêneros. Foram abordados os processos de cuidado em saúde e o direcionamento de políticas públicas. Metade dos estudos apresentou vivências da população transgênero enquanto objeto de estudo. A outra metade tratou de realidades da população LGBTQIAPN+ e diversidade de gênero (Quadro 2). 

**Tabela 1:** Características dos estudos incluídos na revisão sistemática

Estudo	País	Desenho	Idade (anos)	População	Amostra
Adan 2021^26^	Estados Unidos	Qualitativo	≥65	Transgêneros	19
Fabbre 2014^28^	Estados Unidos	Qualitativo	≥50	Transgêneros	22
Fredriksen-Goldsen 2016^39^	Estados Unidos	Seccional	≥50	LGBT	4.627
Rosenwohl-Mack 2022^27^	Estados Unidos	Qualitativo	≥61	LGBTQIA+	21
Pang et al. 2019^29^	Canadá	Qualitativo	≥60	Transgêneros	24
Knochel, Flunker 2021^31^	Estados Unidos	Qualitativo	≥55	Transgêneros	24
Willis 2020^32^	Estados Unidos	Qualitativo	≥50	Transgêneros	19
Muraco 2018^30^	Estados Unidos	Qualitativo	≥50	LGBT	59
Bouman 2016^38^	Reino Unido/Espanha/Bélgica	Seccional	≥50	Transgêneros	71
Hoy-Ellis 2016^36^	Estados Unidos	Seccional	≥50	Transgêneros	186
Hillman 2021^35^	Estados Unidos	Seccional	≥50	Transgêneros	3.462
Blosnich 2016^34^	Estados Unidos	Seccional	21-70+	Transgêneros	6.307
Hol-Ellis et al. 2022^40^	Estados Unidos	Seccional	≥50	LGBT	2.560
Nelson 2023^37^	Estados Unidos	Seccional	≥50	LGBT	1.072
Page 2016^33^	Reino Unido	Qualitativo	Não refere	Transgêneros	Não refere

Entre os estudos qualitativos incluídos, destacam-se: a relação entre usuários não cisheteronormativos e profissionais de serviços responsáveis pela atenção à saúde e as implicações de valores culturais, religiosos e morais no planejamento e execuções dos cuidados em saúde; as particularidades da população LGBTQIAPN+ no planejamento financeiro da velhice; a importância da rede social de suporte; e as particularidades do envelhecimento transgênero diante da transfobia e do idadismo (Quadro 2).

Os estudos abordaram direcionamento de ações específicas para a otimização do processo de envelhecimento da pessoa transgênero. Essas ações foram a oferta de habitações amigáveis, a necessidade de treinamento dos profissionais de saúde sobre a diversidade de gênero, o planejamento de cuidados para o idoso transgênero, incluindo formas de apoio não humanas e o direcionamento de ações públicas, tais como padronização de cuidados e processos de apoio.

A maioria dos estudos quantitativos abordou a temática transgênero enquanto forma de caracterizar o gênero dos sujeitos. Os estudos apresentaram abordagens relativas à situação de saúde do idoso transgênero, com enfoque na saúde mental. Os estudos sobre saúde mental abordaram relações com o estigma da identidade de gênero e a correlação entre o uso hormonal e a saúde mental (Quadro 3).

**Quadro 2:** Estudo da temática transgênero em pesquisas qualitativas

Estudo	Abordagem da saúde	Abordagem da transgeneridade	Objeto do estudo	Conclusão do estudo
Adan 2021^26^	Experiências de vida e percepções de saúde	A vivência da pessoa transgênero é objeto de estudo	Perspectivas de indivíduos transgêneros sobre cuidados de saúde e envelhecimento	Capacitação para os cuidadores de idosos transgêneros é necessária; medo de abusos e sobreposição de estigmas.
Fabbre 2014^28^	Experiências de vida e percepções de saúde	A vivência da pessoa transgênero é objeto de estudo	Transição de gênero entre pessoas idosas	Profissionais podem reproduzir comportamentos que invalidam a velhice *queer*.
Rosenwohl-Mack 2022^27^	Experiências de vida e percepções de saúde	Caracterização do gênero dos sujeitos	Experiência de pessoas idosas LGBTQIA+ residentes em habitação amigável às minorias sexuais e de gênero	Necessidade de oferta de habitações amigáveis a idosos LGBTQIA+.
Pang 2019^29^	Planejamento de cuidados	Caracterização do gênero dos sujeitos	Planejamento de cuidados de vida de adultos LGBT mais velhos	Planejar o cuidado para idosos e garantir que a velhice transgênero seja debatida.
Knochel, Flunker 2021^31^	Planejamento de cuidados	A vivência da pessoa transgênero é objeto de estudo	Planejamento de cuidados na velhice por pessoas transgênero e não binárias	Envelhecimento precoce e necessidade de criação de cuidados prolongados institucionalizados e amigáveis à pessoa idosa transgênero e não binária.
Willis 2020^32^	Cuidado em saúde	Caracterização de gênero dos sujeitos	A interação com profissionais da saúde.	Aprimorar profissionais sobre a temática da diversidade de gênero, a fim de que não reforcem desigualdades com base em gênero a partir da reiteração da cisheteronormatividade.
Muraco 2018^30^	Cuidado em saúde	Caracterização de gênero dos sujeitos	Suporte social de animais entre pessoas idosas LGBT	Diferentes formas de apoio, incluindo as não humanas, podem impactar o envelhecimento LGBT, especialmente os que têm rede social limitada.
Page 2016^33^	Políticas públicas	A vivência da pessoa transgênero é objeto de estudo	Participação social da pessoa idosa transgênero em conselhos de saúde	Foram definidas como ações a identificação de padrões para o cuidado de pessoas transgênero pela equipe de saúde mental e a implementação de processo de apoio aos trabalhadores transgênero.

Estudaram-se a influência dos determinantes sociais na saúde de idosos transgêneros, as investigações acerca do estado de saúde, o impacto da discriminação e da hormonização na saúde mental, a violência conjugal e a relevância de políticas públicas no combate à transfobia (Quadro 3). Não foram identificados dados ausentes ou pouco claro nos estudos incluídos nesta revisão.

**Quadro 3:** Estudo da temática transgênero em pesquisas quantitativas

Estudo	Abordagem da saúde	Abordagem da temática transgênero	Objeto de estudo	Conclusão do estudo
Bouman 2016^38^	Análise da situação de saúde mental	Caracterização do gênero dos sujeitos	Correlação entre uso de hormônios e saúde mental	O uso de hormônios do gênero oposto antes de procurar tratamento médico é generalizado entre mulheres trans mais velhas e parece estar associado a benefícios psicológicos.
Hoy-Ellis 2016^36^	Análise da situação de saúde mental	Caracterização do gênero dos sujeitos; maiores taxas de disforia de gênero	Estigma da identidade de gênero e a correlação com saúde mental	Ao identificar o papel do serviço militar na saúde mental de idosos transexuais, esse estudo fornece *insights* sobre como o serviço militar anterior pode contribuir para a resiliência e resultados positivos em saúde mental.
Blosnich 2016^34^	Análise da situação de saúde	Caracterização de gênero dos sujeitos a partir de cadastros com diagnósticos baseados no CID 9	Prevalência de determinantes sociais da saúde entre veteranos transgêneros e suas associações com condições médicas	Determinantes sociais têm importância na vida de pessoas transgênero; daí a necessidade de registrá-los eletronicamente e incluí-los em metas de tratamento.
Hoy-Ellis 2022^40^	Análise da situação de saúde	Caracterização do gênero dos sujeitos	Diferenças em exames de saúde, conforme a identidade de gênero	Aumentar o acesso dos idosos transexuais à saúde preventiva, por meio de testes de rastreio são fundamentais para reduzir prejuízos de saúde nessa população idosa.
Fredriksen-Goldsen 2016^39^	Experiências de vida e percepções de saúde	Caracterização do gênero dos sujeitos	Eventos da vida e sua associação com bem-estar de idosos	Contextos histórico e ambiental enquadram eventos de vida normativos e não normativos.
Hillman 2021^35^	Análise da situação de saúde	A vivência da pessoa transgênero é objeto de estudo	Violência praticada por parceiro íntimo contra pessoas idosas transgênero	A violência por parceiro íntimo se relaciona a piores níveis de saúde. Recomendação de vigilância que reconheça identidade de gênero e rastreamento de abusos a pessoas +50.
Nelson 2023^37^	Políticas públicas	Caracterização do gênero dos sujeitos	Associação entre políticas públicas e saúde de lésbicas, gays, bissexuais e transgêneros idosos	Adultos lésbicas, gays, bissexuais e transgêneros têm risco significativamente maior de problemas de saúde se viverem em um estado com menos políticas antidiscriminatórias LGBT promulgadas.

## DISCUSSÃO

A produção científica sobre a saúde de idosos transgêneros apresentou notável predominância de estudos norte-americanos. Elencaram-se fatores para justificar as disparidades de produções científicas de países capitalistas desenvolvidos em detrimento de países de média e baixa renda. Tais fatores foram: as desigualdades no investimento em ciência, a presença mais forte da lógica produtivista acadêmica em países ricos, a baixa captação de produções com enfoque nacional por bases de dados internacionais e a possibilidade de discriminação editorial.^20^

Uma pessoa idosa é todo e qualquer indivíduo com 60 anos de idade ou mais.^21^ Há dissenso entre os autores ao estudar o fenômeno das velhices das minorias sexuais e de gênero. A maioria dos estudos elege 50 anos ou mais como ponto de corte, portanto dez anos a menos que o previsto pela Organização Mundial de Saúde. A velhice, permeada pela demarcação cronológica, é uma construção social, e a problematização acerca da demarcação do início deve levar em conta fatores localmente relevantes de cada nação.^22^ A existência marcada pela exclusão social e pela discriminação, especialmente entre pessoas transgênero, tem impactos diretos na qualidade de vida e, consequentemente, na velhice. As particularidades da velhice das minorias sexuais e de gênero são atravessadas pela discriminação e invisibilidade.^23^

Um dos estudos usou por base o diagnóstico de transtorno de gênero, delimitado a partir do código relativo à condição na Classificação Internacional de Doenças, constante no cadastro de saúde do público-alvo. A transexualidade, em um reflexo de hierarquia dos saberes-poderes, reafirmando normas hegemônicas de gênero, tem sido abordada historicamente enquanto desvio, ora de ordem mental, ora de ordem sexual, como demonstra o itinerário das mudanças de categoria no Manual de Diagnóstico e Estatístico de Transtornos Mentais e na Classificação Internacional de Doenças. Os saberes médico, jurídico, psiquiátrico e psicológico incorrem na escolha consciente de patologizar pessoas transgênero, a fim de classificar, diagnosticar e intervir em seus corpos.^24^

A sociedade ocidental é historicamente construída e rotineiramente reforça a ordem cis-heterossexual-patriarcal, que permeia as relações sociais, caracterizadas por uma série de violências, estigmas e discriminações à população transgênero. A reprodução incessante do modelo de performar e viver o sexo e o gênero ocasionam comportamentos trans excludentes.^25^ Os achados desta revisão reiteram que a ordem cisheterossexual de performar gênero e sexualidade atravessa a história de pessoas transgênero. Isso reflete em dificuldades de manutenção de redes sociais de apoio e de acesso ao cuidado qualificado acerca das especificidades de suas velhices. Os comportamentos transfóbicos permanecem ao longo da vida adulta. Na velhice, são acrescidos de elementos que caracterizam o contexto de vida da população idosa, como a exclusão familiar e o receio de comportamentos discriminatórios por parte de cuidadores e demais moradores de instituições de longa permanência.

Destaca-se a heterogeneidade dos estudos incluídos, com desenhos diversos, o que dificultou a generalização dos resultados, como fator limitante desta revisão.

A temática transgênero nas pesquisas sobre a saúde do idoso tem sido estudada a partir da perspectiva do acúmulo de estigmas e da continuidade de discriminações e exclusões durante a velhice. Destaca-se o receio da solidão, da continuidade da transfobia e da baixa oferta espaços abertos e acolhedores a idosos transgêneros, culminando em uma rede social de apoio frequentemente mais fragilizada.

A importância desta revisão se justifica pela relevância social do tema, dado o atual cenário de envelhecimento populacional. As velhices que se contrapõem à cisheteronormatividade exigirão de profissionais e sistemas de saúde o conhecimento das particularidades e vivências de idosos LGBTQIAPN+, em especial, idosos transgêneros. A ausência de estudos que correlacionem o envelhecimento às minorias sexuais e de gênero na América Latina e no Caribe oportuniza a análise contextualizada das vivências de idosos transgêneros no continente.
